# Effect of brick kiln emissions on commonly used vegetables of Kashmir Valley

**DOI:** 10.1002/fsn3.252

**Published:** 2015-06-25

**Authors:** Bhat M. Skinder, Afeefa Q. Sheikh, Ashok K. Pandit, Bashir A. Ganai, Aashiq H. Kuchy

**Affiliations:** ^1^ Centre of Research for Development/Department of Environmental ScienceUniversity of KashmirSrinagarIndia

**Keywords:** Biochemical parameters, *Brassica oleracea*, brick kiln, emissions, *Phaseolus vulgaris*, *Solanum melongena*

## Abstract

To study the impact of brick kiln emissions on plant growth and productivity, a study was conducted on various biochemical parameters of three main vegetables *Brassica oleracea* L., *Phaseolus vulgaris* L., and *Solanum melongena* L. cultivated in the vicinity of the brick kiln area of the Panzan village of district Budgam (J&K). Plants in the vicinity of brick kilns are direct recipients of emissions and therefore important materials for assessing potential effects of kiln pollutants. The biochemical values of all the three vegetables of the brick kiln site when compared to the control site are significantly (*P* ≤ 0.05) different. The findings of the present work depict that the brick kilns are the prime reason for the deterioration of important consumable vegetables, which could lead to chaos in the food security of the area in concern besides a threat to local people in terms of health if proper pollution control devices or the replacement of brick kilns are not put in place with new technology.

## Introduction

Plants are indispensable part of ecosystems and their sensitivity to air pollution is more considerable than standards of air pollution (Thomas 1991). Air pollution has become a serious environmental stress to crop plants due to increasing industrialization and urbanization during the last few decades (Rajput and Agrawal 2004). Diverse changes induced by different air pollutants in plants with respect to morphological, anatomical and physiological characteristics have been investigated (Rao 1981; Pawar and Dubey 1983; Rao and Dubey 1988). The most dispersed and injurious pollutants in industrial areas (brick kilns) are sulfur dioxide (SO_2_), nitrogen oxides (NO_x_), carbon monoxide (CO), tropospheric ozone (O_3_), and heavy metals, as well as suspended particulate matter (Asgher and Singh, ; Assadi et al. 2011). Estimates indicate that annual emissions from a brick industry were 80 tons of particulate, 30 tons of carbon, 7 tons of NO_x_, and 5 tons of SO_x_ (Asgher and Singh, 2003). A range of air pollutants is recognized as phytotoxic agents and phytotoxicity of sulfur dioxide has been documented for about a century (Godzik and Sienkiewicz 1990), sound effects of ozone for more than 30 years (Miller 1983), acidic precipitation for more or less 20 years (Likens et al. 1979), and effects of prominent levels of nitrogen compounds, nitrogen oxides, and ammonia in the last decade (Nihlgard, ). The significance of other pollutants such as peroxy acetyl nitrate (PAN) (Su et al. 2006), fluorides (Maclean 1981), or heavy metals have also been documented (Unsworth and Harrison 1985). Plants in the immediate vicinity of emissions sources are more vulnerable. It has been revealed that the most sensitive species of plants start on to show visible signs of damage to sulfur oxides at concentrations of about 1850 μg/m^3^ for 1 h, 500 μg/m^3^ for 8 h, and 40 μg/m^3^ for the maturing season (Smith 1981, cited in NAPAP 1990). Sulfur dioxide shows negative effects in terms of foliar injury, physiological and biochemical alterations on vegetation (Ganai et al. 2007a,b; Balkhi et al. 2009; Irshad et al. 2011), and short‐term treatment of SO_2_ damaged PSII, decreased the fluidity of thylakoid membrane, and affected the process of electron transport (Liu et al. 2007). The particulates and gaseous pollutants, alone and in combination can cause grave setbacks to the overall physiology of plants (Ashenden and Williams 1980; Mejstrik 1980; Anda 1986). Of all plant parts, the leaf is the most sensitive part to the air pollutants (Singh 1990).

Accumulation of particulates on the surface of the plants can in due course alter plant vulnerability to pathogens and pests (Emberson et al. 2001) and the exposure to dust provoked a significant reduction in photosynthesis in most plants. Thus, may alter plant growth and production, without physical damage to the plant (Kumar and Thambavani 2012). It is now generally accepted that cellular membranes are among the primary sites of pollutant attack and, since lipids are important membrane components and play essential roles in maintaining membrane structure and function, many workers have examined the effects of pollutants on lipids to clarify the mechanisms of their phytotoxicity (Mudd et al. 1984; Sakaki 2002). The fact that plants provide a vast leaf area for impingement, assimilation and accumulation of air pollutants diminish the pollutant level in the air environment (Warren 1973; Shannigrahi et al. 2004), it is reported that depending on their sensitivity level, plants show visible changes which would include alteration in the biochemical processes or accumulation of certain metabolites (Agbaire and Esiefarienrhe 2009), thus can be used as bioindicator of air pollution (Tripathi and Gautam 2007; Lalitha et al. 2013).

Brick kilns are known to be a leading cause of ambient air pollution in rural areas. The levels of SO_x_, NO_x_, and SPM are major problems in the Panzan village of district Budgam because the levels of these pollutants are significantly higher than NAAQS guidelines during the operational phase of brick kilns and especially play a significant role in elevating the SO_2_ concentration in the ambient air, as they are fed with sulfur rich, inferior quality of coal (2.5 to 3.0 tons/season/kiln) besides the use of inefficient and outdated technology such as Bull's Trench kilns (Skinder et al. 2014). High air pollution levels in the atmosphere have shown the significant negative importance, as they do not only pose serious occupational health hazards but also adversely affect the surrounding environment (Guttikunda 2009; Ganai et al. 2010; Pawar et al. 2010).

Considering the facts that air pollutants have damaging effects and shocking consequences on the exposed plants, the present work was designed to estimate the biochemical parameters of the commonly used vegetables of Kashmir (India). The study evaluated the effects of brick kilns on the biochemical parameter and the consequences there upon. Our study highlighted the concern of brick kiln emissions on the quality of local vegetables which is highly a pressing issue among the people living in the study area and other similar populations throughout the globe.

## Material and Methods

This study was conducted in the rural area of district Budgam in Kashmir valley at Panzan village, where mushrooming of brick kilns is a serious threat to air quality and consequently to vegetation. For the plant analysis, the fresh leaves of three available vegetables were used as study material and were collected from two sites, that is, brick kiln site (Site I) (app. 50 m away, 33°57′56.51″N and 74°46′19.06″E) and the other site with similar ecological conditions was selected as the control (least polluted) Site II (app. 2–4 km away, 33°58′38.66″N and 74°48′43.33″E) throughout the growing season over a period of 3 months from July 2012 to September 2012 during the operational phase of brick kilns. The leaf samples were immediately taken to the laboratory for analysis.

### Biochemical analysis

Fresh leaves of the vegetables were used as study material and were collected from two sites, that is, brick kiln site (S I) and control site (S II). The samples of plant species were collected in replicates, placed in airtight polyethylene bags separately and carried to the laboratory for further analysis. The material was washed thoroughly and moisture was drained before analysis for various biochemical contents. The analysis was done as per standard methods:

### Estimation of total protein

The protein content of vegetable leaves was determined colorimetrically by the method of Lowry et al. (1951) after the extraction of proteins with buffer solution of pH‐7.

One gram (1 g) of fresh weight of plant material was homogenized by grinding with buffer solution of pH‐7 using mortar and pestle. About 0.2 mL and 0.4 mL of the extract were pipetted into two separate test tubes and 5 mL of freshly prepared alkaline copper tartrate reagent was added, followed by 0.5 mL of IN Folin's reagent to each test tube. The contents of each test tube were immediately vortexed and subsequently incubated for 30 min at room temperature for color development. The absorbance was then read at 700 nm against a reagent blank using visible spectrophotometer. Bovine serum albumin was used as standard and the results were expressed as mg% of fresh weight of leaves.

### Estimation of total carbohydrates

The carbohydrate content of vegetables was determined colorimetrically by phenol–sulfuric acid method after extraction into the buffer solution of pH 7 (Dubois et al.*,* 1951).

One gram (1 g) of fresh weight of plant material was homogenized with buffer solution of pH‐7 using mortar and pestle. About 0.5 mL and 1 mL of the extract were pipetted into separate test tubes and 1 mL of 5% phenol was added followed by rapid addition of 5 mL of conc. H_2_SO_4_. The contents were shaken thoroughly and subsequently incubated for 40 min at room temperature for color development. The absorbance was then measured at 490 nm against a reagent blank using visible spectrophotometer. Glucose was used as standard and the results were expressed as mg% of fresh weight of leaves.

### Estimation of total lipids

The total lipid content of the vegetables was determined colorimetrically by Sulphophosphovanillin method after extraction in buffer solution of pH‐7 (Knight et al. 1972).

One gram (1 g) of fresh weight of plant material was homogenized with buffer solution of pH‐7 in pestle mortar and 0.1 mL of the extract was pipetted in a separate test tube and digested with 2 mL of conc. H_2_SO_4_. After cooling the test tube, 5 mL of Phosphovanillin reagent was added and the sample was subsequently incubated for 40 min at room temperature for color development. The absorbance was then measured at 540 nm against a reagent blank using visible spectrophotometer. Olive oil was used as standard and the concentration of total lipid was calculated by using the following formula.

**Calculation:**

$$\left(A_{T}-A_{B}\right)/\left(A_{S}-A_{B}\right)\;\times \;500,$$

where *A*_T_ = Absorbance of test sample; *A*_B_ = Absorbance of blank; *A*_S_ = Absorbance of standard. The results were expressed as mg percentage of fresh weight of vegetables leaves.

### Estimation of photosynthetic pigments chlorophyll, phaeophytin, and carotenoids content in photosynthetic tissue (leaf)

The method of plant pigment isolation and estimation from green plant leaves is based on the principle of extraction of loosely protein‐bound pigments by the help of organic solvents such as acetone and measuring their color intensity in visible region by Spectrophotometric method.

One gram (1 g) of fresh weight of the main chlorophyll‐bearing organ of the plant species was extracted with 80% aqueous acetone by macerating the samples using a mortar and pestle. The decanted suspension was centrifuged for 3 min at 195 g. After centrifugation, the upper green clear solution was decanted from the colorless residue and then made up to 10 mL with 80% acetone in 10 mL test tubes. The material was then subjected to centrifugation at 11200 g for 10 min. The optical density (O.D) of the solution was determined using a spectrophotometer at different wavelengths like 665 nm, 649 nm, 666 nm, 510 nm, and 480 nm, respectively. The results were expressed as μg/mL of fresh weight of plant sample.

1. The chlorophyll content was measured according to Strain et al. 1971
  Chlorophyll a (μg/mL) = 11.63 (O.D.665) – 2.39 (O.D.649)
  Chlorophyll b (μg/mL) = 20.11 (O.D.649) – 5.15 (O.D.665)
  Total Chlorophyll (μg/mL) = 6.45 (O.D.665) + 17.72 (O.D.649)
2. The phaeophytin content was estimated according to Vernon (1960)
  Phaeophytin a (μg/mL) = 20.15 (O.D.666) – 5.87 (O.D.665)
  Phaeophytin b (μg/mL) = 31.96 (O.D.665) – 13.65 (O.D.666)
  Total phaeophytin (μg/mL) = 6.75 (O.D.666) + 26.03 (O.D.665)
3. The carotenoids content was estimated according to method of Duxbury and Yentsch (1956)
  Carotenoids (μg/mL) = 7.6 (O.D.480) – 1.49 (O.D.510)

### Statistical analysis

All experiments were performed in triplicates. Statistical analysis were performed using Statistical Package for Social Sciences (SPSS 16.0) software for Windows with a significance level of alpha = 0.05. One‐way ANOVA and the Duncan's new multiple range test (Sokal and Rohlf 1981) was used to compare the means as well as the seasonal differences in various parameters in control and also in the vegetable leaves affected by brick kiln emissions.

## Results and Discussion

The data collected for two sites (Site I and Site II), were compared to find out the impact of brick kiln emissions on plant productivity. *Brassica oleracea* (locally called Saagh) showed a general decrease in respect of total chlorophyll, total phaeophytin, carotenoids, protein, carbohydrate, and lipids as the exposure level of pollutants increases in due course of time at the brick kiln site (Site I) till it touched the lowest (74.36%, 74.27, 51.99, 55.85, 53.12, and 28.31%, respectively, in all parameters) in the month of September (Table 1).

**Table 1:** Impact of brick kiln emissions on biochemical parameters of *Brassica oleracea* L.

Parameters	July			August			September		
	S‐II (Control)	S‐I	%D	S‐II (Control)	S‐I	%D	S‐II (Control)	S‐I	%D
Chlorophyll ‘a’ (μg/mL)	22.68 ± 0.90^d^	17.33 ± 0.64^c^	23.59	22.14 ± 1.32^d^	11.35 ± 1.85^b^	48.75	20.75 ± 1.61^d^	5.66 ± 0.16^a^	72.70
Chlorophyll ‘b’ (μg/mL)	18.08 ± 3.04^d^	12.10 ± 2.46^bc^	33.10	13.11 ± 1.17^bc^	9.55 ± 2.59^b^	27.16	14.50 ± 0.7^c^	3.37 ± 0.15a	76.73
Total Chlorophyll (μg/mL)	40.76 ± 2.23^e^	29.42 ± 3.09^c^	27.81	35.25 ± 2.48^d^	20.90 ± 2.10^b^	40.72	35.25 ± 1.00^d^	9.04 ± 0.13^a^	74.36
Phaeophytin ‘a’ (μg/mL)	29.65 ± 3.8^d^	21.16 ± 2.00^c^	28.65	27.31 ± 2.47^d^	14.50 ± 1.22^b^	46.88	25.97 ± 0.7^d^	4.53 ± 0.15^a^	82.55
Phaeophytin ‘b’ (μg/mL)	42.99 ± 1.75^d^	33.34 ± 3.71^c^	22.44	41.71 ± 3.42^d^	21.89 ± 4.06^b^	47.53	39.49 ± 4.35^d^	12.34 ± 0.38^a^	68.75
Total Phaeophytin (μg/mL)	73.04 ± 2.58^e^	54.78 ± 1.70^c^	24.99	69.39 ± 4.10^de^	36.59 ± 4.43^b^	47.27	65.81 ± 3.66^d^	16.93 ± 0.38^a^	74.27
Carotenoids (μg/mL)	14.13 ± 0.45^e^	11.42 ± 1.50^c^	19.22	13.51 ± 0.91^de^	8.91 ± 0.24^b^	34.04	12.14 ± 0.33^cd^	5.83 ± 0.34^a^	51.99
Proteins mg%	41.78 ± 3.97^de^	38.89 ± 4.20^cd^	6.94	45.94 ± 4.81^e^	31.28 ± 2.58^b^	31.92	47.03 ± 4.30^e^	20.77 ± 1.41^a^	55.85
Carbohydrates mg%	60.15 ± 4.71^c^	57.64 ± 4.76^c^	4.18	63.85 ± 3.15^c^	43.95 ± 4.12^b^	28.79	67.05 ± 3.09^d^	31.44 ± 3.62^a^	53.12
Lipids mg%	10.81 ± 0.76^bc^	9.45 ± 0.83^a^	12.62	12.01 ± 1.00^c^	9.80 ± 0.72^ab^	18.43	15.34 ± 0.85^d^	11.00 ± 0.49^bc^	28.31

Data represent the mean of three replicates analyzed separately ±standard deviation values and %D = percent of decrease; Values with different superscripts in rows are significantly (*P* ≤ 0.05) different.

A similar trend was maintained by other two vegetables (*Phaseolus vulgaris* L. and *Solanum melongena* L.) with increasing level of exposure of pollutants at the brick kiln site (Site I). The highest decrease in investigated biochemical parameters like total chlorophyll, total phaeophytin, carotenoids, protein, carbohydrate, and lipids was observed in the month of September again. However, there were some remarkable differences in the levels of said parameters of the vegetables. In case of *Phaseolus vulgaris* L., the decreased percent were 61.37, 55.30, 55.58, 64.58, 68.59, and 14.26% in the respective biochemical parameters when compared to the control site (Site II) (Table 2). While as, in case of *Solanum melongena* the decreased levels were 60.56, 49.18, 28.78, 63.66, 60.99, and 11.94% for total chlorophyll, total phaeophytin, carotenoids, protein, carbohydrate, and lipids, respectively, for Site I as compared to the control Site II (Table 3).

**Table 2:** Impact of brick kiln emissions on biochemical parameters of *Phaseolus vulgaris* L.

Parameters	July			August			September		
	S‐II (Control)	S‐I	%D	S‐II (Control)	S‐I	%D	S‐II (Control)	S‐I	%D
Chlorophyll ‘a’ (μg/mL)	24.35 ± 0.33^d^	19.50 ± 0.67^c^	19.92	23.69 ± 0.50^d^	14.82 ± 1.38^b^	37.45	20.60 ± 0.53^c^	10.06 ± 0.62^a^	51.17
Chlorophyll ‘b’ (μg/mL)	20.20 ± 0.55^e^	16.70 ± 1.35^cd^	17.31	17.85 ± 0.87^d^	8.49 ± 0.54^b^	52.44	14.93 ± 2.43^c^	3.67 ± 0.15^a^	75.44
Total Chlorophyll (μg/mL)	44.55 ± 0.70^e^	36.20 ± 1.44^c^	18.74	41.54 ± 1.14^d^	23.31 ± 0.89^b^	43.89	35.52 ± 2.75^c^	13.72 ± 0.71^a^	61.37
Phaeophytin ‘a’ (μg/mL)	32.96 ± 0.65^d^	22.34 ± 2.81^b^	32.23	30.61 ± 0.27^cd^	19.99 ± 0.51^b^	34.69	28.88 ± 2.02^c^	9.16 ± 1.45^a^	68.29
Phaeophytin ‘b’ (μg/mL)	45.63 ± 1.17^d^	39.45 ± 3.02^c^	13.55	44.85 ± 1.50^d^	26.67 ± 3.70^b^	40.53	37.27 ± 1.91^c^	20.46 ± 2.37^a^	45.12
Total Phaeophytin (μg/mL)	79.02 ± 0.82^e^	62.08 ± 1.57^c^	21.44	75.87 ± 1.33^e^	46.93 ± 3.19^b^	38.15	66.53 ± 2.17^d^	29.74 ± 1.32^a^	55.30
Carotenoids (μg/mL)	14.18 ± 0.17^c^	11.95 ± 0.46^b^	15.76	12.14 ± 0.50^b^	5.57 ± 0.39^a^	54.09	14.13 ± 0.75^c^	6.28 ± 0.34^a^	55.58
Proteins (mg %)	49.83 ± 2.92^c^	50.91 ± 3.05^d^	−2.17	57.01 ± 3.61^e^	31.02 ± 2.95^b^	45.60	63.89 ± 3.46^e^	22.63 ± 3.51^a^	64.58
Carbohydrates (mg %)	62.99 ± 2.54^c^	58.47 ± 2.96^c^	7.17	71.89 ± 4.16^d^	50.00 ± 3.73^b^	30.44	73.92 ± 4.31^d^	23.22 ± 3.10^a^	68.59
Lipids (mg %)	68.59 ± 3.91^ab^	65.86 ± 4.35^a^	3.98	72.16 ± 3.25^bc^	68.71 ± 3.26^ab^	4.79	84.27 ± 2.48^d^	72.25 ± 3.03^c^	14.26

Data represent the mean of three replicates analyzed separately ±standard deviation values and %D = percent of decrease; Values with different superscripts in rows are significantly (*P* ≤ 0.05) different.

**Table 3:** Impact of brick kiln emissions on biochemical parameters of *Solanum melongena* L.

Parameters	July			August			September		
	S‐II (Control)	S‐I	%D	S‐II (Control)	S‐I	%D	S‐II (Control)	S‐I	%D
Chlorophyll ‘a’ (μg/mL)	21.74 ± 1.56^d^	20.60 ± 2.01^d^	5.24	24.57 ± 0.99^e^	12.56 ± 1.96^b^	48.88	17.60 ± 0.42^c^	9.20 ± 1.24^a^	47.73
Chlorophyll ‘b’ (μg/mL)	13.10 ± 4.55^bc^	11.37 ± 0.70^b^	13.19	17.58 ± 2.75^d^	9.59 ± 0.76^b^	45.44	15.65 ± 1.24^cd^	3.92 ± 0.84^a^	74.99
Total Chlorophyll (μg/mL)	34.84 ± 6.04^c^	31.98 ± 2.17^c^	8.23	42.15 ± 3.70^d^	22.15 ± 1.21^b^	47.45	33.25 ± 0.94^c^	13.11 ± 0.89^a^	60.56
Phaeophytin ‘a’ (μg/mL)	29.19 ± 2.20^d^	23.82 ± 3.20^c^	18.39	34.09 ± 2.15^e^	17.09 ± 2.72^b^	49.85	20.53 ± 1.42^bc^	12.14 ± 1.38^a^	40.86
Phaeophytin ‘b’ (μg/mL)	39.42 ± 5.24^cd^	39.64 ± 3.05^cd^	−0.57	44.63 ± 1.89^d^	23.22 ± 2.91^b^	47.97	35.53 ± 0.70^c^	16.33 ± 2.04^a^	54.05
Total Phaeophytin (μg/mL)	69.00 ± 5.45^d^	63.79 ± 6.19^cd^	7.56	79.17 ± 4.01^e^	40.54 ± 5.66^b^	48.79	56.34 ± 1.19^c^	28.63 ± 3.44^a^	49.18
Carotenoids (μg/mL)	19.16 ± 1.78^c^	15.80 ± 1.59^b^	17.54	20.97 ± 2.31^c^	13.39 ± 0.08^ab^	36.16	15.96 ± 0.87^b^	11.36 ± 1.82^a^	28.78
Proteins (mg %)	77.65 ± 2.40^d^	65.49 ± 3.93^c^	15.66	84.50 ± 4.07^e^	48.04 ± 2.64^b^	43.15	85.12 ± 4.63^e^	30.93 ± 2.57^a^	63.66
Carbohydrates (mg %)	61.72 ± 3.60^c^	59.89 ± 2.48^c^	2.97	65.89 ± 5.10^c^	49.38 ± 3.85^b^	25.06	71.83 ± 4.29^d^	28.02 ± 2.18^a^	60.99
Lipids (mg %)	161.46 ± 2.50^c^	148.25 ± 2.25^a^	8.18	166.32 ± 3.43^c^	149.11 ± 4.00^a^	10.35	175.09 ± 3.22^d^	154.19 ± 4.17^b^	11.94

Data represent the mean of three replicates analyzed separately ±standard deviation values and %D = percent of decrease; Values with different superscripts in rows are significantly (*P* ≤ 0.05) different.

The photosynthetic pigments are the most liable to be damaged by air pollution. Chlorophyll is said to be an index of productivity, hence any alteration in chlorophyll concentration may change the morphological, physiological, and biochemical behavior of the plant. From the analysis of the results of chlorophyll pigments, a considerable reduction was found at all the polluted sites compared to the control site during the entire study period. Results have shown the negative impact of brick kiln emissions on chlorophylls of the vegetable species namely *Brassica oleracea*,* Phaseolus vulgaris* L., and *Solanum melongena* L. At Site I (brick kiln site), a general decrease was observed in chlorophyll content as compared to Site II (Control site). This is probably due to the exchange of gaseous SO_2_ which causes direct injury to crops by entering the leaves through the stomata (Heather 2003) and dry or wet deposition of dust, fly ash, SO_2_, and NO_2_ on the leaves causing reduced interception of incident light and clogging of stomata (Chauhan and Joshi 2010). Investigations conducted elsewhere have shown that SO_x_, NO_x_, CO, and fly ash cause gross destruction of the thylakoid membrane system in the chloroplast (CLAG, 1996; Wellburn 1998; Liu et al. 2007). Injuries in the thylakoid are likely to be connected with a decline in the amount of chlorophyll (Malhotra and Hocking 1976). Pollutants can cause leaf injury, stomatal damage, premature senescence, and can decrease photosynthetic activity, disturb membrane permeability, and reduce growth and yield in sensitive plant species (Agrawal and Deepak 2003; Agrawal et al. 2006; Tiwari et al. 2006; Dwivedi and Tripathi 2007). The brick kiln emissions adversely affects the surrounding vegetation as the plants are exposed not to only one but too many air pollutants. Rao and Leblanc (1966) mentioned that high amount of gaseous SO_2_ causes destruction of chlorophyll and that might be due to the replacement of Mg^+2^ by two hydrogen atoms and degradation of chlorophyll molecules to phaeophytin. Mg^++^ is replaced by two molecules of hydrogen with a resulting change in the light absorption spectral properties of chlorophyll molecules.

A significant decrease in phaeophytin, carotenoids, proteins, carbohydrates, and lipid content was also noted in the samples collected at Site I (Brick kiln site) as compared to control site (S II). This could be attributed to brick kiln pollution stress as had confirmed by Ganai et al. (2010). Also phaeophytin and carotenoids content decreased on exposure to sulfur dioxide (Balkhi et al. 2009; Irshad et al. 2011). Carotenoids guard from photo‐oxidation damage; hence, their decrease has serious outcomes on chlorophyll pigments (Sifermann and Harms 1987). The decrease in protein concentration could be attributed to inactivation of enzymes due to air pollutants. At the higher concentration of pollutants like SO_2_ near brick kilns probably break enzymes and other proteins, enhanced the rate of protein denaturation, a fact being supported by the findings of Prasad and Inamdar (1990) and Constantinidou and Kozlowski (1979).

Carbohydrates are important constituent and source of energy for all living organisms. Plants manufacture this organic substance during photosynthesis and break down during respiration (Tripathi and Gautam 2007). In this study, carbohydrates in polluted leaves were reduced under pollution conditions. This could be fairly due to the destruction of photosynthetic pigments as a result of brick kiln emissions. These results are in consonance with the findings of Ganai et al. (2010). The concentration of carbohydrate is indicative of the physiological activity of a plant and it determines the sensitivity of plants to air pollution. Reduction in carbohydrate content at polluted site (Site I) can be attributed to increased respiration and decreased CO_2_ fixation because of chlorophyll deterioration (Tripathi and Gautam 2007). Davison and Barnes (1986) mentioned that pollutants like SO_2_, NO_2_, and H_2_S under hardening conditions can cause more depletion of carbohydrates in the leaves of plants grown in polluted area. The reaction of sulfite with aldehydes and ketones of carbohydrates can also cause reduction in carbohydrate content. The decrease in carbohydrate also could be due to the destruction of chlorophyll which adversely affects the rate of photosynthesis because of the competition between CO_2_ and SO_2_ for the carboxylase enzyme (Ziegler 1973).

Unlike other parameters’ lipids in samples collected at Site I (Brick kiln site) showed increasing trend with the increase in time duration, but lipid content was not increasing with such pace as in control samples. It is believed that lipids get accumulated when the growth is restricted during the autumn season (cold season) and were consumed during the growth period, that is, in summers (Meletiou‐Christou et al. 2011). Subsequently, when lipid content of Site I was compared to control samples (Site II), the reduction was seen in all the samples of vegetables. This is because of increasing pollution load in due course of time. According to detailed ultrastructural observations of plant cells injured by these air pollutants, cellular membrane systems are affected by the pollutants (Thomson 1975; Huttunen and Soikkeli 1984; Tiwari et al. 2006) and membrane permeability is also seen to change after treatment or exposure to SO_2_ and other pollutants (Malhotra and Hocking 1976; Mudd et al. 1984; Sakaki 2002).

## Conclusions

Results have shown the negative impact of brick kiln emissions on biochemical parameters of the three vegetables *Brassica oleracea*,* Phaseolus vulgaris* L., and *Solanum melongena* L. Thus, the results of the current study provide evidence that brick kiln emissions are a significant risk to the vegetable plants in the Panzan area of district Budgam. As a result, it depicts that brick kilns are the prime reason for the deterioration of important consumable vegetables, which could lead to both food as well as health concern among the local population. Also, this will be a serious threat to millions of people depending on the vegetables grown near such kiln emission areas. The imbalance in the biochemical constituents in vegetables will lead to serious consequences on the overall living standard of exposed people in the near future if mushrooming of brick kilns is not controlled or replaced with new technology.

## Conflict of Interest

None declared.
